# Advancing digital health: FDA innovation during COVID-19

**DOI:** 10.1038/s41746-020-00371-7

**Published:** 2020-12-17

**Authors:** Kushal Kadakia, Bakul Patel, Anand Shah

**Affiliations:** grid.417587.8 0000 0001 2243 3366 U.S. Food and Drug Administration, 10903 New Hampshire Avenue, Silver Spring, MD 20993 USA

**Keywords:** Health policy, Clinical trial design

## Abstract

Digital health products have played an important role in the COVID-19 response, from supporting the remote monitoring of patients to enabling continuity in data collection for clinical trials. The U.S. Food and Drug Administration (FDA) has issued a number of temporary policies to support digital health innovation during the pandemic, such as guidance documents to expand the use of digital therapeutics for psychiatric disorders and medical devices for remote patient monitoring. In this article, we contextualize these policies to the agency’s existing regulatory framework for digital health, outline key considerations for patients and health care providers, and identify implications for the future of digital health innovation.

The COVID-19 pandemic has accelerated the transition to digital health in the American health care system. For example, many clinical trial sponsors have adopted telehealth and remote patient monitoring technologies to enable continuity in data collection during the pandemic. Likewise, various mobile medical applications and software functions have been used to support public health surveillance, enable the dissemination of educational materials, and streamline communication for patients and providers. Furthermore, regulatory relief from the Centers for Medicare & Medicaid Services (CMS) has enabled clinicians to shift visits to virtual platforms to reduce infection risk to patients.

The Food and Drug Administration (FDA) has issued multiple temporary policies to support the uptake of these tools during the public health emergency^1^. These actions are an extension of the agency’s longstanding commitment to advancing regulatory science for digital health, which was articulated in 2017 with the Digital Health Innovation Action Plan and solidified with the creation of a Digital Health Center of Excellence, which was launched in 2020^2,3^. In this article, we contextualize FDA policies to support digital health during the COVID-19 pandemic to the agency’s existing risk-based approach to regulation, and preview the implications of these policies for the future of digital health innovation.

## FDA’s regulatory framework for digital health

When Congress provided FDA with the authority to regulate medical devices in 1976, medical technologies were largely analog^4^. Hardware-based devices differ significantly from software-based devices in terms of their design, development life cycle, and risk-benefit calculus. As innovators began to develop digital tools to reduce care fragmentation, promote personal wellness, and support the diagnosis and treatment of disease, the agency recognized the need to develop a regulatory framework that stays current and is attuned to the unique considerations of digital tools ^5^.

Of note, software functions that do not meet the definition of a medical device in the Federal Food, Drug, and Cosmetic (FD&C) Act are outside of the agency’s device regulatory purview. For example, the FDA does not regulate videoconferencing platforms that are used to enhance communication between patients and providers—such as the virtual modalities used for clinical visits during COVID-19—because they do not meet the device definition. Digital health products that are medical devices, such as mobile medical applications used to diagnose irregularities in cardiac rhythm, are regulated by FDA according to the level of risk posed to consumers.

To provide momentum for the digital transformation of American medicine, the FDA issued policy guidance in 2013 and subsequently updated it in 2015 and 2019^6^. Early progress from the FDA’s Digital Health Innovation Action Plan was promising and established risk-based policies explaining FDA’s regulatory approach. For example, the agency has focused its oversight on higher-risk mobile medical applications (e.g., those used to diagnose or treat patients), but not on lower-risk digital health products (e.g., those focused solely on promoting wellness)^2^. As part of the Digital Health Innovation Action Plan, the FDA is also exploring the creation of a Software Pre-Certification program, which is currently in development for software as a medical device as a pilot^7^. By developing an oversight process and mechanisms that take into account a developer’s capability, a streamlined review of the product’s analytical and clinical performance, and the products’ real-world performance, the agency believes that a regulatory model as envisioned in the Software Pre-Certification Pilot program can be designed to keep pace with digital health advances and provide a reasonable assurance of safety and effectiveness.

## Digital health innovation for COVID-19

Several milestones demonstrate how thoughtful regulatory strategies can advance the development of digital health products. For example, the FDA cleared the first-ever digital health therapeutic in 2018, and has cleared several products since then, most recently a game-based therapeutic for attention deficit hyperactivity disorder^8,9^. Additionally, the agency has continued to iterate on its Software Pre-Certification Pilot program, publishing an update on initial lessons in 2020^10^.

However, the onset of the COVID-19 pandemic has dramatically and rapidly increased the value proposition of digital health, with unprecedented adoption and utilization of new software tools and digital platforms by payers and providers to meet patient needs during the public health emergency. The FDA has sought to expand access to clinically-appropriate, low-risk digital health tools during the COVID-19 pandemic by stating its intention not to enforce certain regulatory requirements for some devices.

A clear use case of regulatory flexibility is mental health care. Research indicates the pandemic has taken a toll on the wellbeing of many Americans, with the number of adults reporting symptoms of psychological distress more than tripling in April 2020 compared to April 2018^11^. To address the enhanced mental health burden, the FDA issued guidance to temporarily expand patient access to digital health therapeutics for psychiatric disorders^12^. Under this policy, FDA stated its intention not to object to the distribution and use of such devices (e.g., computerized behavioral therapy, mobile medical applications for mental health) for the duration of the public health emergency without the submission of a premarket notification under Section 510(k) of the FD&C Act or compliance with other requirements, such as those for Unique Device Identification.

Likewise, experts are concerned about the health consequences of pandemic-induced interruptions to chronic disease management^13^. Consequently, the FDA issued guidance stating that it does not intend to object to limited modifications to the indications, claims, functionality, or hardware or software of certain non-invasive remote monitoring devices (e.g., blood pressure measurement systems) that are used to support patient monitoring during the public health emergency to help reduce the risk of infection from in-person clinical visits^14^.

It is important to emphasize that flexibilities do not compromise the agency’s continued prioritization of patient safety and product quality. As noted in FDA guidance, the temporary policies issued for certain digital health products during the COVID-19 pandemic are limited to select devices with low-risk profiles that can offer meaningful benefit to patients (e.g., therapeutics for psychiatric disorders). For example, the agency has provided specific examples in its guidance on remote patient monitoring devices to explain when device modifications would not present an undue risk (e.g., changes in parameter display) versus device modifications that do present an undue risk (e.g., functional changes to signal acquisition systems). These actions and scenarios highlight the value of a risk-based approach to regulation, which allows the FDA to be responsive to evolving public health needs by adopting policies for certain low-risk devices, while using agency resources for the evaluation of high-risk products. Of note, several of the software functions relevant for public health officials during the pandemic—such as the use of mobile applications for contact tracing—are not medical devices and therefore do not require FDA device review.

## Regulatory anticipation of risks and challenges

When considering regulatory flexibilities during a public health emergency, the FDA must carefully weigh the need for expedited access to timely innovations with the potential risks to consumers. In the context of digital health during the COVID-19 pandemic, the agency has identified two potential areas for concern.

First, part of the value proposition of digital health is the potential for such technologies to help bridge informational siloes in the traditionally fragmented American health care system. Low-risk digital health products, such as certain devices for remote patient monitoring, could offer unique insights to providers to improve care management. However, it is important to ensure that enhanced access to such innovations does not encroach upon a patient’s right to personal privacy, especially with industry increasingly designing new digital tools for use in home environments as opposed to traditional care sites. Standards for the use and disclosure of protected health information by certain entities are set forth in regulations implementing the Health Insurance Portability and Accountability Act of 1996 (HIPAA). FDA recognizes that complying with HIPAA is an important part of an innovator’s device design.

Second, if data on the utilization and performance of digital health products during the pandemic are not collected and shared, then it will limit the agency’s ability to learn from the COVID-19 experience to inform future regulation. The FDA has previously highlighted its commitment to advancing the use of real-world evidence to support regulatory decision-making for medical devices, and the significant quantities of real-world data generated from the increased utilization of digital health technologies during the pandemic provide an opportunity to expand the evidence base for benefit-risk determinations and patient preferences^15^. Indeed, the collection of real-world performance data are key components of the FDA’s working model for its proposed Software Pre-Certification program^16^. Consequently, innovators would also do well to use this opportunity and carefully collect data, in a responsible way, on the patient experience with these products during the COVID-19 pandemic and share such insights with the agency. Doing so would help inform regulators’ understanding of the performance of digital products in different settings and among diverse populations, and understand the risk tolerance of both patients and physicians to use such tools in various clinical scenarios.

## Implications for the future of digital health regulation

While the pandemic remains ongoing, it is already evident that COVID-19 will have a lasting impact on health care delivery. Regulatory flexibilities have been an important enabler of change during the pandemic; however, guidance and policies issued during the pandemic—including those for digital health—are temporary. FDA intends to rigorously review these temporary policies as part of its Pandemic Recovery and Preparedness Plan, which seeks to identify opportunities for long-term reforms^17,18^. Digital health will be a cornerstone of this effort, and agency officials expect emerging evidence to inform regulatory science for several key areas.

First, the pandemic will provide valuable insights about the utilization of and experience with digital health products. Data from payers and developers can provide population-level context about the utilization of technologies such as devices for remote patient monitoring. Meanwhile, feedback from providers and patients can offer experiential insights, from risk tolerance to the compatibility of the user interface. These perspectives can inform how agency officials might adjust the agency’s regulatory framework for digital health products, from determinations of product risk to the proper framework for a future Software Precertification Program.

Second, research has shown that the uptake of digital health products among elderly Americans has generally lagged due to issues such as the accessibility of product design^19^. However, the pandemic provides an opportunity to accelerate the adoption of digital health technologies among older patients, who may be more likely to now embrace virtual care delivery to minimize their comparatively higher risk for infection. For example, CMS data indicates that the number of Medicare beneficiaries using telehealth services increased from 13,000 to 1.7 million during the COVID-19 pandemic^20^. If similar trends are observed for medical devices (e.g., digital health therapeutics, remote patient monitoring technologies), then the resulting performance data in this population can help harmonize evidence standards for digital health products between the FDA and CMS—a longstanding interagency priority. As regulation of digital health products evolves, payers will need to modernize reimbursement systems to support a world in which the locus of care delivery is increasingly shifted away from the hospital and towards the home. For example, CMS last year issued reimbursement policies for remote patient monitoring (e.g., of physiological parameters such as pulse oximetry). Such services could be integrated into care models such as hospital-at-home programs^21^.

Third, the use of digital health tools in clinical trials (e.g., wearables to measure vital signs, mobile applications to measure patient adherence) was already increasing prior to the pandemic, with more 1100 trials using a connected digital health product in 2018^22^. COVID-19 has provided further momentum for this trend, with pandemic-induced disruptions to trial operations (e.g., delayed patient visits) leading some investigators to adapt study processes using digital tools^23^. To enable continuity in data collection without compromising patient safety, the FDA issued a guidance document outlining best practices for trial sponsors, including the use of remote patient monitoring and telehealth, when appropriate^24^. Understanding the experience of investigators and study participants with digital health products can help to inform future regulatory guidance, particularly as clinical trials become more decentralized and sponsors work to digitize processes ranging from enrollment to data collection.

The pandemic has accelerated the arrival of the digital era in many aspects of American health care. The FDA will continue to learn and iterate to advance regulatory science for digital health advancement in accordance with emerging evidence and stakeholder feedback, remaining committed to its goal of supporting patient-centered innovations that are safe and effective.
